# ‘Distancers’ and ‘non‐distancers’? The potential social psychological impact of moralizing COVID‐19 mitigating practices on sustained behaviour change

**DOI:** 10.1111/bjso.12399

**Published:** 2020-06-25

**Authors:** Annayah M. B. Prosser, Madeline Judge, Jan Willem Bolderdijk, Leda Blackwood, Tim Kurz

**Affiliations:** ^1^ Department of Psychology University of Bath UK; ^2^ Department of Psychology Faculty of Behavioural and Social Sciences University of Groningen the Netherlands; ^3^ Department of Marketing Faculty of Business and Economics University of Groningen the Netherlands; ^4^ School of Psychological Science University of Western Australia Perth Australia

**Keywords:** COVID‐19, social distancing, social identities, shaming, behaviour change, moralization, Covidiots

## Abstract

COVID‐19 mitigating practices such as ‘hand‐washing’, ‘social distancing’, or ‘social isolating’ are constructed as ‘moral imperatives’, required to avert harm to oneself and others. Adherence to COVID‐19 mitigating practices is presently high among the general public, and stringent lockdown measures supported by legal and policy intervention have facilitated this. In the coming months, however, as rules are being relaxed and individuals become less strict, and thus, the ambiguity in policy increases, the maintenance of recommended social distancing norms will rely on more informal social interactional processes. We argue that the moralization of these practices, twinned with relaxations of policy, may likely cause interactional tension between those individuals who do vs. those who do not uphold social distancing in the coming months: that is, derogation of those who adhere strictly to COVID‐19 mitigating practices and group polarization between ‘distancers’ and ‘non‐distancers’. In this paper, we explore how and why these processes might come to pass, their impact on an overall societal response to COVID‐19, and the need to factor such processes into decisions regarding how to lift restrictions.

## Background

Latest reports in the United Kingdom suggest social distancing measures will continue for the remainder of 2020 (Mason & Proctor, 2020), and the likelihood of returning to ‘normality’ appears low. While compliance with COVID‐19 mitigating practices (such as wearing masks or socially distancing) has been high in most countries (Hale *et al*, 2020), it remains to be seen whether this holds as the requisite changes become more permanent and disruptive to daily life (Walker *et al*, 2020).

As tight regulations around social distancing relax, and immediate threat decreases, interpersonal differences will likely come to the fore: Some individuals will continue practising stringent measures under reduced policy interventions, whereas others will not. Such interpersonal differences, partly fuelled by government communications, may be perceived as arising from differences in underlying moral values (Everett *et al*, 2020). Some see it as their moral duty to keep socially distancing, in order to protect vulnerable fellow citizens from COVID‐19 infection (Lewnard & Lo, 2020). Others may disagree, believing that such actions are immoral and will harm the economy, increasing unemployment and societal harm in different ways (Sample, 2020). Furthermore, individuals will likely experience dilemmas between their duty to adhere to social distancing ‘rules’, and obligations to provide sufficient care for vulnerable individuals in their community (e.g., visiting isolated elderly people, supporting protests, or supporting grieving friends). Thus, as we move into a long‐term response to the crisis, COVID‐19 mitigating practices are becoming increasingly moralized around different concerns and in multiple contexts (e.g., physical or mental health, economic and social). These competing moralizations of mitigating practices possess a high potential for interpersonal disagreement among the public regarding the ‘right’ practices to adopt moving forward (Kurz, Prosser, Rabinovich & O'Neill, 2020).

Prior research suggests moral disagreement is a particularly painful and hostile form of disagreement (Haidt, Rosenberg & Hom, 2003). When both sides of the spectrum feel their particular preference is the morally correct course of action, the other party is not just ‘different’ but ‘wrong’ (Goodwin & Darley, 2012). As COVID‐19 mitigating behaviours (such as social distancing or mask wearing) are so strongly seen as morally relevant practices (Francis & McNabb, 2020), their continued enactment as regulations ease means potentially walking on *socially* thin ice. Perceived moral motivation for behaviour, regardless of specific content of the moral arguments, is enough to produce defensiveness and self‐concept threat in others, which may lead individuals to ‘double‐down’ on their own moralized behaviour and derogate others who behave differently (Cramwinckel, van Dijk, Scheepers & van den Bos, 2013). In this way, the mere *potential* of a moral motivation for a practice is enough to invoke *perceptions* of judgement and outrage towards those who behave differently.

Moralization processes can be shaped by multiple sources. Government communications function as moralized persuasion contributing to the interpretation of new moral norms in society (e.g., Rozin, 1999; Täuber, 2018). Moral norms can also be collectively negotiated informally in social interactions between individuals and within communities, as seen in community efforts to dissuade visitors to rural areas (Murphy, 2020). Moralization has been described as a double‐edged sword, functioning as a powerful motivator of behavioural change, but also producing defensiveness and interpersonal difficulties (Kovacheff *et al*., 2018; Minson & Monin, 2012; Täuber, 2019). We argue that these two ingredients of moralization and emerging disagreement about whether to maintain mitigating practices, form a potent recipe for social interactional trouble, do‐gooder derogation and group polarization. In this paper, we provide an overview of some of the potential downstream consequences of moralizing COVID‐19 mitigating practices, focusing primarily on group processes and how their potential negative impacts should be factored into decision‐making surrounding continued policy relaxation.

## What are the social consequences of moralizing practices?

Although moral intuitions and decision‐making are commonly investigated at the individual level, morality also plays a role in regulating the behaviour of individuals within groups (Ellemers & van den Bos, 2012; Ellemers, Pagliaro, & Barreto, 2013). At the intragroup level, during COVID‐19 people are likely interpreting and negotiating novel moral norms within their ingroups (i.e., at the interpersonal, community, or national level). This involves attempting to regulate others’ behaviour with regard to these norms, for example, expressing disapproval of perceived moral violations (Steentjes, Kurz, Barreto & Morton, 2017). Thus far, social sanctioning has been largely directed at individuals who do not keep social distance (Tait, 2020). It is, however, conceivable that as measures relax and voices rejecting perceived restrictive norms grow louder, people start socially (and economically) sanctioning those who stubbornly keep social distance, despite reduced threat and relaxed regulations. Moreover, individuals anticipating a future weakening of restrictions may try to get ahead of the curve and may feel comfortable relaxing their own behaviours before it is officially advised (Sparkman & Walton, 2017). This effect could be particularly acute where early relaxation may confer significant economic or social advantages (e.g., reopening a business, celebrating an elderly family member’s birthday). So too, when societal authorities (e.g., politicians and public health officials) fail to give clear and consistent signals in line with scientific advice: or violate policies themselves without consequence. This introduces further ambiguity to policies and undermines public trust, confidence and overall adherence to mitigating practices (Curtis, 2020; Dandekar & Barbastathis, 2020; Jetten, Reicher, Haslam & Cruwys, 2020).

These intragroup processes may result in negative interpersonal consequences for people striving to maintain social distancing guidelines, such as anticipated moral reproach and ‘do‐gooder derogation’ (i.e., backlash against individuals *perceived* as claiming moral superiority; Monin, 2007; Minson & Monin, 2012). Concerns about potential derogation in this context may reduce public adherence to recommended mitigating measures (Lin, Schaumberg & Reich, 2016). This form of derogation may impact already‐vulnerable groups, such as the elderly or immunocompromised, and increase interactional trouble for those who choose to continue socially isolating. One could imagine a scenario where an individual is socially ostracized by friends for refusing to attend a social gathering or challenged for avoiding important in‐person workplace meetings. This ostracism could manifest itself through accusations of ‘poor manners’ or ‘lack of support’, where an individual’s own COVID‐mitigating practices are newly translated by others into perceived social or indeed moral affronts. Thus, previously neutral behaviours have moved into newly moralized domains (Francis & McNabb, 2020) and as such can develop social consequences (Williams, 2020) that may hasten intergroup conflict and polarization.

The willingness and ability of some individuals to maintain and advocate continued mitigating practices are a key component of many governments’ plans to ease strict lockdown measures (WHO, 2020). Despite the importance of these individuals, we argue that without the support of formal rules and regulations, social interactional trouble may begin to plague these actors, who may be perceived as ‘moral rebels’ (e.g., Monin, Sawyer & Marquez, 2008) and incur significant social and psychological costs. Additionally, negotiations of moral norms at the community level can also be complicated by moral dilemmas (e.g., concerning one’s caring responsibilities) and this can be one site where moralized identities emerge and clash.

At the intergroup level, it may not yet be obvious that groups are involved in these processes, since the initial process of moralization typically occurs prior to any recognizable identity labels (Täuber, 2019). Once a norm is generally accepted, however, the non‐compliant may be viewed as a disruptive minority of ingroup deviants who may be symbolically 'ejected' from the group and assigned a stigmatizing label (Hales, Ren & Williams, 2017). This was evident when, at the height of the pandemic, those who did not adhere closely to existing guidelines (e.g., by travelling to second homes or gathering with friends) were stigmatized as ‘non‐distancers’ or ‘covidiots’ (Iqbal & Townsend, 2020). Conversely, as lockdown measures relax, there may be people who for moral or pragmatic reasons are unable to return to ‘normal’ (e.g., those who are immunocompromised), who may then be stigmatized as 'distancers'.

Finally, both these minority positions have the potential to be re‐appropriated as a self‐adopted identity (e.g., ‘I'm a proud Distancer’), which can further contribute to ostracism. Paradoxically, members of ‘extreme’ groups may be even more likely to attract ostracism if their group is perceived to be pro‐social rather than antisocial (e.g., Hales & Williams, 2019). Thus, moralization of mitigating practices may ultimately lead to new ingroup and outgroup identities based on adherence to, or flouting of, newly negotiated moral norms; and to group polarization.

Recent work (Kurz *et al*, 2020) highlights some of the difficulties faced by those promoting wide‐scale adoption of practices that are ‘moralized’ on account of their environmental and other collective benefits. They point to potential perils associated with the creation of (practice‐based) social identities around these behaviours (e.g., ‘Vegetarians’), stemming from a range of emergent social interactional processes such as ‘Do‐gooder derogation’ (Minson & Monin, 2012). Moreover, they suggest these processes can render such social identities sticking points to the achievement of societal‐level tipping points in rates of practice adoption. At its peak, COVID‐19 arguably represented the inverse of that social psychological dynamic. We have seen the immediate appearance of institutionally endorsed normative behavioural practices enacted by the majority in pursuit of collective (public health) benefit (Jetten, Reicher, Haslam & Cruwys, 2020). As such, there was initially limited opportunity for social identities to form around moralized practices and for conflict to emerge between groups enacting different practices. We are now looking ahead to a period where divergences in people’s interpretations of and behavioural responses to official guidance may occur. At the height of the pandemic, people were not disparagingly referred to in social discourse as a ‘hand‐washer’ or ‘distancer’. As the crisis lengthens and becomes ‘the new normal’, however, the kinds of social identity dynamics so commonly observed within other moralized practice domains are also likely to make an appearance.

An important factor in how people interpret and respond to guidance will be people’s different lived experiences of social isolation. These different experiences will in part reflect how people are positioned structurally (e.g., in terms of gender, race, and class‐based social identities: EuroHealthNet, 2020). There may be significantly more social interactional tension inherent in a low‐paid worker in the gig economy seeking to minimize social contact in the face of relaxed guidelines, compared to a high‐paid office worker who can more easily maintain social distancing without negative consequences. Thus, the ability to perform, let alone, to moralize one’s own behavioural practices may not be equally distributed, and these inequalities may strengthen polarization in emergent identities. Moreover, there is considerable potential for these moralized social identities to politicize. The beginnings of this process have been seen in ‘anti‐lockdown’ protests around the world (e.g., Italy, the United States, South Africa) around various causes including unequal access to food, medicines, and PPE; defence of the economy and concerns about increasing unemployment; and the protection of individual rights and civil liberties (Burke, 2020). While some of these protests were directed at ending the requirement to socially distance, others focused on the failure of governments to facilitate people’s social distancing practices (e.g., through providing PPE and addressing inequalities in disease incidence). As lockdown measures ease and attention turns to the devastation of people’s lives and communities, unrest and the further politicization of both distancing and non‐distancing identities appear likely.

## How could individuals, communities, and policymakers address these issues effectively?

In this section, we discuss social psychological insights that may be useful for considering how to best support vulnerable people in our communities over the longer term.

### What individuals can do?

#### A gentle approach to interpersonal norm negotiation

Novel moral norms around social distancing are currently being negotiated across multiple countries and contexts, and debates and argumentation are likely to be a common element of these negotiations. These processes can be intensified in online contexts, where it is not possible to read other peoples’ non‐verbal cues. Although ostracism and stigmatization are commonly used strategies to influence the behaviour of others in one’s community, research suggests this is a painful experience that may push people towards identifying with other people who also feel excluded and may even contribute to identity polarization (Hales *et al*., 2017; Täuber, 2019). Stigmatization can also sometimes inadvertently punish individuals who have no choice but to engage in the target behaviour (e.g., an immunocompromised person wearing a mask, in a context where masks are rare). We would encourage people to be careful of the attributions they make and to focus on the best way to collectively and respectfully resolve moral dilemmas as they arise. Groups in our communities can also play their part through support and collective advocacy to ensure social support for the vulnerable (e.g., mutual aid) and to ensure that those who need to socially distance in their homes, communities, and workplaces have the means to do so (e.g., trade unions).

### What policymakers can do?

#### Consider the role of moral content in persuasive messaging

Our analysis suggests that it may be important to consider when it is appropriate to use moral or non‐moral messaging to encourage adherence to social distancing practices. Moral content can be important for creating drastic, rapid behaviour change (van Bavel *et al*., 2020); however, in the longer term, moral messages may contribute to social interactional difficulties and polarization (Täuber, 2019). An alternative or complementary strategy is to use non‐moral or pragmatic messages (Kreps & Monin, 2014; Täuber, Van Zomeren & Kutlaca, 2015). Such messages could communicate that mitigating practices have become ‘our new normal’; that is, they are widely accepted in the community and do not necessarily signal individual moral virtue. A cautiously optimistic (albeit slightly disquieting) prediction could be that some practices (e.g., substitutes for handshaking) will become solidified as ‘simple common‐sense’ thus stripped of significant moral connotations.

#### Maintain institutional signals

Through signals of what represents the default, institutions can influence behaviour, without officially enforcing it (Tankard & Paluck, 2016). The 1.5‐m spaced floor markings that have appeared in many public spaces signal that distancing is considered important (i.e., an injunctive norm). Although organizations may want to remove these markers, our analysis suggests benefits in their retention; it may help motivated and vulnerable individuals to uphold and protect social distance in public settings, and avoid potential social backlash by others for doing so. Institutional signals (including physical cues within the environment) could help individuals avoid moral disagreement and thus social interactional trouble.

#### Consider dynamic norms signalled by policy changes

In addition to signalling current injunctive norms, it is important to consider how institutional signals may also imply a dynamic descriptive norm (e.g., Sparkman & Walton, 2017). Ongoing relaxation of government restrictions could unintentionally imply that the worst has passed, and society is on a trajectory back to ‘normal’. This may increase perceptions that only a ‘minority’ are still engaging in social distancing, accentuating the social difficulties we have discussed. Avoiding this impression is important as it may lead to people relaxing their practices faster than the guidelines suggest (i.e., pre‐conformity with an anticipated future norm) which in turn communicates a more relaxed descriptive norm. Rather than rolling back restrictions in a stepwise fashion that implies an incremental return to ‘normal’, consideration could be given to relaxing the rules via qualitative steps that still imply a degree of caution is needed. Those engaged in the difficult task of modelling behavioural outcomes of removing restrictions would also do well to consider such dynamic‐norm‐signalling effects.

## Conclusion

In summary, the moralization of COVID‐19 mitigating practices, combined with relaxation of regulations, may have significant unintended consequences that impact people’s ability to maintain practices and social cohesion. We believe the processes described above are of interest to the social psychological community and should be studied further. COVID‐19 is an exceptional circumstance, but analysis of this moment may prove useful for the study of other rapid social change efforts surrounding moralized practices, such as the climate crisis (Reese *et al*, 2020).

A critical factor in how well governments and other authorities (e.g., police, health workers, employers) manage dynamically loosening regulations is their relationship of mutual trust and respect with all communities (Bradford *et al*, 2020; De Cremer & Blader, 2006). As lockdown eases internationally, there will be more ambiguity and different normative practices might emerge for different groups and contexts. Recognition of these challenges in norm negotiation and expressions of trust from authorities are critical: It communicates that people are regarded as a valued community member and as moral citizens capable of making moral choices (Renger & Simon, 2011; Tyler & Blader, 2000). Moving forward, what is seen as moral or normative will be very dynamic and contextually based. Authorities and individuals must ensure their constructions of what is normative and moral mesh with people’s lived experiences and their other identity positions. To prevent unintended consequences in the relaxation of social distancing measures, individuals, communities, and policymakers should avoid essentializing others as ‘moral’ or ‘immoral’. Instead, we should support dynamic norm negotiation, and empower people to safely enact practices that protect the health and well‐being of themselves and their community, in the face of potential social interactional and personal difficulties.

## Conflict of interest

All authors declare no conflict of interest.

## Author contribution

Annayah Miranda Beatrice Prosser (Conceptualization; Investigation; Project administration; Writing – original draft; Writing – review & editing) Madeline Judge (Writing – original draft; Writing – review & editing) Jan Willem Bolderdijk (Supervision; Writing – original draft; Writing – review & editing) Leda Blackwood (Supervision; Writing – original draft; Writing – review & editing) Tim Kurz (Supervision; Writing – original draft; Writing – review & editing).

## Data Availability

No data were collected for this brief report.
